# Honey proteins regulate oxidative stress, inflammation and ameliorates hyperglycemia in streptozotocin induced diabetic rats

**DOI:** 10.1186/s12906-023-03837-9

**Published:** 2023-01-18

**Authors:** Farwa Naqvi, Nida Dastagir, Almas Jabeen

**Affiliations:** 1 Dr. Panjwani Center for Molecular Medicine and Drug Research, International Center for Chemical and Biological Sciences, University of Karachi, Karachi, 75270 Pakistan; 2 Dow University of Health Sciences (DUHS), Karachi, Pakistan

**Keywords:** Diabetes, Inflammation, Honey Proteins, Immunomodulatory, *Ziziphus* honey

## Abstract

**Background:**

Diabetes Mellitus (DM) poses a serious health problem worldwide and several inflammatory mediators are involved in the pathogenesis of this disease. Honey composed of various constituents which have been proven to have immunomodulatory and anti-inflammatory properties. The aim of this study is to investigate the in vitro and in vivo effects of *Ziziphus* honey and its isolated crude proteins in modulation of immune system and inflammation involved in the pathogenesis of diabetes.

**Methodology:**

The proteins from *Ziziphus honey* were isolated by ammonium sulfate precipitation and estimated by Bradford method. In vitro anti-inflammatory activities were evaluated by inhibition of reactive oxygen species (ROS) from phagocytes *via* chemiluminescence immunoassay and nitric oxide (NO) by Griess method. Cytotoxicity was evaluated by MTT Assay. The comparative effect of oral and IP routes of honey and isolated proteins was observed in streptozotocin (STZ) induced diabetic male Wistar rats. qRT-PCR technique was utilized for gene expression studies.

**Results:**

The honey proteins suppressed phagocyte oxidative burst and nitric oxide (NO) at significantly lower concentrations as compared to crude honey. The isolated proteins showed promising anti-inflammatory and hypoglycemic effects along with maintenance of body weight of rodents *via* both oral and IP routes, with significant down-regulation of inflammatory markers TNF-α, IL-1β, IFN-γ, iNOS, caspase 1, Calgranulin A (S100A8) and NF-κB expression in diabetic rats.

**Conclusion:**

The isolated honey proteins showed better immunomodulatory and therapeutic potential at significantly lower doses as compared to crude honey.

## Background

Diabetes Mellitus (DM) is categorized as one of the frequently occurring disease worldwide, in which chronic hyperglycemia occurs due to insufficient production of insulin that causes metabolic disturbances of carbohydrate, lipid and protein [1, 2]. It is among one of the most prevalent disease which is the leading cause of mortality and morbidity [3]. International Diabetes Federation (IDF) presented statistical report in 2015, concluding 415 people were having diabetes and the number may be raised to 642 million by 2040. Pakistan, among one of the developing countries, is buried under socioeconomic burden of diabetes and, having prevalence rate of 22% in urban and 17% in rural areas [4]. Unhealthy lifestyle and high rise in urbanization, contributes to the increase risk of diabetes. Other factors including hereditary, reduced physical activity, age related problems and obesity also played major role in its increased incidence. Inflammation is among one of the alarming factors of diabetes which has been gaining interest in the field of research [5].

Various inflammatory processes are involved in the development of both types of diabetes [6]. An elevation in the levels of anti-inflammatory and pro-inflammatory cytokines has been observed in both pre-diabetic as well as diabetic patients [7, 8]. Abnormal increment of TNF-α and IL-6 in adipose tissue leads to insulin resistance in type 2 diabetes [9] whereas production of IFN-γ, IL-1β and TNF in type 1 diabetes cause toxic effects on beta cells [10, 11]. IL-1β along with TNF-α and IL-6 can induce apoptosis in pancreatic beta cells through the downstream activation of NF-κB or JNK pathway [10]. Caspase-1 is also responsible for activation of pro-inflammatory cytokines including IL-18 and IL-1β [12]. Presence of high levels of S100A8/9 is also associated with the pathogenesis of diabetes [13] and this protein complex can be used as a biomarker [14].

Natural products have varying level of biological and therapeutic potential as well as immunomodulatory properties. According to global estimation more than 50% of the natural products are used as drugs from centuries [15]. Products obtained from honey bee including propolis, royal jelly, and honey are known to have immunomodulatory potential [16]. Traditionally, bee products have been used in medicine from the ancient times [17]. Honey act as immunomodulator and possess both immunostimulatory and immunosuppressant activities. It stimulates the cytokine production by immune cells, which contributes in disease treatment [18, 19]. However, the major hindrance in understanding the properties of honey and optimizing its clinical applications is the lack of knowledge of its components responsible for immunoregulation and, its origin from vast variety of floral population. The floral honey constituents include carbohydrates along with proteins, water, enzymes, organic acids, vitamins and phytochemicals. Natural honey having low protein content, which widely varies, according to floral variety. Characterization of honey protein can reveal floral and geographical origin of honey [20]. Previous studies shown the potential of honey protein as an immunomodulatory agent [21]. MRJP1 a glycoprotein in honey stimulates the production of TNF-α in mice macrophages and the effect was majorly contributed by its protein moiety that justify the interactions between honey glycoproteins and glycopeptides with immune system [22].

*Ziziphus* (Sidr) (also known as Lote tree, Christ's Thorn, Jujube or Nabkh tree, botanical name: *Ziziphus spina*-christi) tree belongs to Rhamnaceae family having about 50 species around the globe. All of its parts have medicinal values including leaves, flowers, stem and seeds. Its leaves are used as disinfectant, for curing swollen eyes, abscesses, and leaves extracts have shown antibacterial, antiviral, and antidiabetic properties. Its seeds are rich in protein and fruits have high energy value. The root and stem bark also used in medicinal preparations. Due to its medicinal properties, taste and fragrance there is high demand of sidr flower honey [23, 24]. The source of honey alters its properties and effects its composition, nutritional values and therapeutic properties. The floral source, climate at the time of harvest and soil composition are also among the key factors in determining the quality and effectiveness of honey for medicinal or health-promoting purposes, therefore the *Ziziphus* honey is selected for present study.

The available medications alone failed to control diabetes effectively, unable to prevent pancreatic beta cell destruction, having complications related to oxidative stress and other adverse effects including weight gain [25]. Honey has shown hypoglycemic effect on streptozotocin (STZ) and alloxan induced type 1 and 2 diabetic models [26, 27]. The reduction in lipid content of serum in diabetic model along with hypoglycemic effect adds the effectiveness of honey [28].

**Fig. 1:**
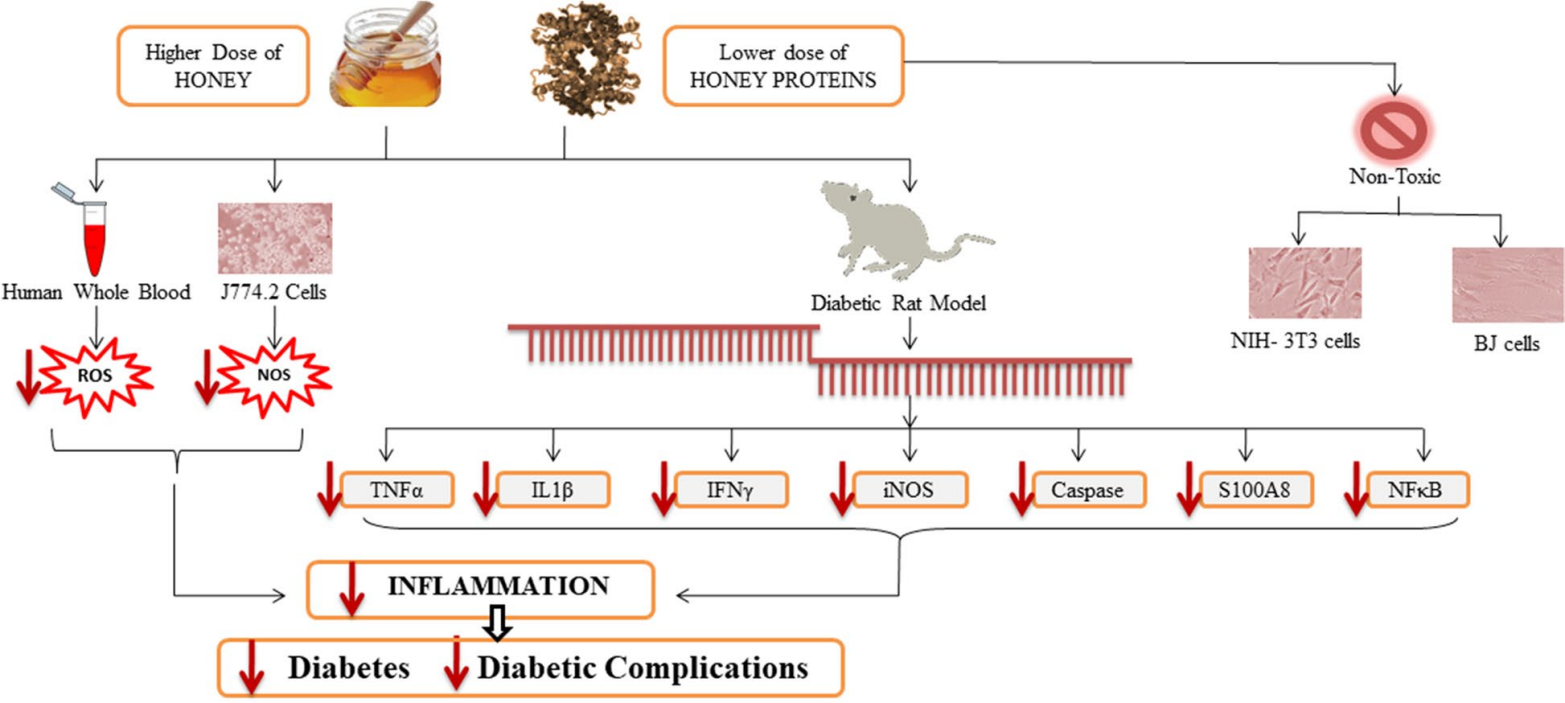
Study design and its expected outcomes

We are involved in further exploring the targeted therapeutic potential of honey which is being traditionally used for its beneficial properties as well as its constituents specifically isolated proteins*.* Previously our colleagues reported the characterization and in vitro immunomodulatory potential of glycoproteins and glycopeptides from *Ziziphus* honey [21]. In continuation of previous in vitro studies, here in we report the in vivo immunomodulatory and hypoglycemic potential of *Ziziphus* honey and its isolated proteins in streptozotocin induced diabetic rats (Fig. 1).

## Materials and methods

Pure, fresh, amber colored *Ziziphus* honey was selected for this study, which was obtained from the northern areas of Pakistan.

### Isolation of honey proteins

Equal volume of honey and tris–HCL were mixed using magnetic stirrer for 5 min, then spun to remove pollens at 2500xg for 25 min at 4**˚**C. Supernatant was collected and its total volume was measured, while pellet was discarded. The quantity of ammonium sulfate to be used for 80% saturation of total protein was estimated, and added to the mixture slowly to precipitate honey at 0ºC. After complete dissolution, and after overnight incubation at 4ºC, the whole mixture was spinned at 8300xg at 4**˚**C for 20 min. The pellet was washed with Tris–HCl buffer solution by spinning at 3000xg for 25 min at 4**˚**C. Finally, the supernatant was carefully collected which contain the proteins, isolated from honey in a crude form. Protein was estimated using Bradford Assay [29, 30].

### In vitro assays

The studies on cells from human blood were carried out after approval from an independent ethics committee, ICCBS, UoK,No: ICCBS/IEC-008-BC-2015/Protocol/1.0. in accordance with the declaration of Helsinki (1964) with the amendments of Tokyo (1975) and Edinburgh (2000). The cell lines used in the study were obtained from Bio bank facility PCMD, ICCBS, UoK.

### Chemillumenescence Immunoassay for Quantifying Intracellular Reactive Oxygen Species (ROS)

The effect of honey and its crude isolated proteins on luminol-enhanced ROS production was quantified using human whole blood which was collected from healthy volunteers of 25–30 years of age [31]. 25 μL of whole blood (1:20 dilution in sterile Hanks Balanced Salt Solution, containing calcium chloride and magnesium chloride (Gibco, USA) was mixed with 25 μL of test samples and incubated for 10 min in luminoskan chamber (Luminoscan RS, Labsystem, Helsinki, Finland). Honey and its proteins were tested at various concentrations (honey at 3.12 μg/mL-100 mg/mL and protein at 0.4 -400 ng/mL). Blood cells were stimulated with addition of 25 μL of serum opsonized zymosan (Wako, China) and intracellular ROS production was detected by adding 25 μL of luminol (Alfa Aesar, Germany). Both activator and detector were added in positive controls while the negative control contained cells and detector only. The plate was placed in the luminoskan chamber and measurements for luminescence as RLU were taken for 50 min with repeated scan mode.

### Nitric oxide assay

The effect of honey and its proteins on NO production was analyzed using mouse macrophage cell line, J774.2 (ECACC, UK). DMEM media (Sigma, USA) supplemented with 10% FBS (Sigma, Canada) and, 1% penicillin/streptomycin (Gibco, USA) was used to culture cells. Upon reaching 70–80% confluency, the cells were harvested using cell culture grade scrappers and centrifuged at 400xg for 6 min at 25 ͦC. The pellet was resuspended in 1 mL of media. Cells were counted in hemocytometer (Marienfeld, Germany) by trypan blue assay. 150 μL of 1 × 10^6^ cells/mL was added along with different dilutions of honey and protein (honey 0.1–100 μg/mL and protein 0.4–400 ng/mL) and 30 μg/mL of LPS (Difco, USA) which was used as an activator. Plate was incubated for 48 h at 37ͦ C in 5% CO_2_. Griess Method was used to determine nitrite accumulates in the supernatant. The absorbance was read at 550 nm using spectrophotometer (Spectra Max plus 340) [32].

### MTT cytotoxicity assay

MTT (3-[4,5-dimethylthiazol-2-yl]-2,5-diphenyl tetrazolium bromide) assay was performed as described by [33] to test in vitro cytotoxicity of honey proteins. NIH-3T3 and BJ (ATCC, Manassas, USA) were used for toxicity assay upon 75% confluence cells were harvested by trypsinization, pelleted at 400xg for 5 min at RT, and resuspended in 1 mL of DMEM. The cell count was adjusted to 6000 cells/well were seeded in 96 well flat bottom culture plates and incubated in 5% CO_2_ at 37ºC. After 24 h, media was removed. 50 μL/well of different dilutions (400 ng, 40 ng, 4 ng, 0.4 ng) of proteins were added in triplicates along with 150µL of DMEM (Sigma, USA). The control wells received 200 µL of media. The plate was incubated for 48 h in the CO_2_ incubator (NU 8700, Nauire Autoflow, Plymouth, USA) at 37ͦ C. The media was removed from wells and 50 μL of 0.5 mg/mL MTT dye (Amresco, USA) was added in each well with 150 μL of media. After 4 h incubation, MTT dye was removed and 100 $\mu$L of DMSO (Fisher Scientific, USA) was added in each well to dissolve formazan crystals. Absorbance was read at 540 nm using spectrophotometer. % Inhibition values at different concentrations of compounds were obtained by the formula, which were used to achieve concentration that showed 50% growth inhibition (IC_50_) by MS Excel based formula.

$$\%\;Inhibition=100\;-\;({OD}_{\;sample}\;-\;{OD}_{\;blank})/\;({OD}_{\;control}\;-\;{OD}_{\;blank})\;\times\;100$$

### In vivo assays

#### Induction of diabetes in rats

Animal procedures were performed on male Wistar healthy rats. Total 42 rats were used in the study weighing 220–250 gms, with the approval of ethical committee of Dr. Panjwani Center for Molecular Medicine and Drug Research, International Center for Chemical and Biological Sciences (ICCBS) (Protocol number 2016–0040), in accordance with the ICCBS Animal care and Laboratory use, issued by (The National Academics, Washington, DC) and ARRIVE guide lines. Prior to induction of diabetes, the animals were fasted overnight and 55 mg/kg of STZ (dissolved in 0.1 M citrate buffer), was administered intraperitoneal (IP). After 1 week, fasting glucose was measured using glucometer by pricking tail vein. The animals, with blood glucose level of 200 mg/dL or higher were considered as diabetic and included in study.

### Treatment protocol

Animals were randomly allocated into 7 groups with *n* = 6 in each group including; normal control receiving distilled water (NC), diabetic control receiving distilled water (DC), diabetic rats receiving honey (2 g/Kg) orally (HO), diabetic rats receiving honey (2 g/Kg) intraperitoneally (HI), diabetic rats receiving honey proteins (1 mg/Kg) orally (PO), diabetic rats receiving honey proteins (1 mg/Kg)intraperitoneally (PI) and diabetic rats receiving standard anti-inflammatory drug indomethacin intraperitoneally (IND) (2 mg/Kg) once daily for 4 weeks.

### Sample collection

After one-month treatment, animals were anesthetized by intraperitoneal administration of (50 mg/kg) sodium pentobarbital for the collection of blood. The 8–10 mL of blood was collected from renal vein and animals were euthanized by cervical dislocation [58].

### RNA isolation and cDNA synthesis for qRT-PCR

RNA from blood collected from animals was isolated with RNA isolation kit (Thermofisher Scientific, Waltham, US). RNA quantification was done using Thermo Scientific nanodrop 2000 spectrophotometer. 1000 ng/1 μg of RNA was used to synthesize complementary DNA (cDNA) by using Revert Aid First Strand cDNA Synthesis Kit (Thermo Fisher scientific, US).

#### Quantitative real time polymerase chain reaction

To evaluate the immunomodulatory activity of honey and its isolated crude proteins, the expression analysis of various inflammatory genes carried out by using Maxima SYBR Green (Thermofisher scientific, United states) using Mx3000P QPCR System (Agilent Technologies, USA). Primers including Nuclear factor kappa B (NF- κB) forward primer 5'-CCCGAAATCAAAGACAAGGAGG-3', reverse primer 5'-CTGTGTTGGATTTAGTGGCTCC-3', Tumor necrosis factor alpha (TNF-α) forward primer 5'-CCACGTCGTAGCAAACCACCAAG-3', reverse primer 5'- CAGGTACATGGGCTCATACC-3', Caspase 1 forward primer 5'- GAGCTTCAGTCAGGTCCATCA-3', reverse primer 5'-TCTGAGGTCAACATCAGCTCC-3', Interferon-gamma (INF-γ) forward primer 5'-GCCCTCTCTGGCTGTTACTG-3', reverse primer 5'-CCAAGAGGAGGCTCTTTCCT-3', Interleukin 1 beta (IL-1β) forward primer 5'- AGGCTTCCTTGTGCAAGTGT-3', reverse primer 5'-ATCTTTTGGGGTCTGTCAGC-3', S100A8 forward primer 5'-TGCCCTCTACAGGGATGACT-3', reverse primer 5'- TCGAAGTTAATTGCGTTGTCA-3', and inducible nitric oxide synthase (iNOS) forward primer 5'- CAATAACCTGAAGCCCGAAG-3', reverse primer 5'- TCTGTGCTGAGAGTCATGGAG-3' and Hypoxanthine phosphoribosyl transferase 1 (HPRT) forward primer 5'-CTTTGCTGACCTGCTGGATT-3', reverse primer 5'-CCCGTTGACTGGTCATTACA-3' was used as a house keeping gene, were designed by obtaining the sequence from the website https://www.ensembl.org/index.html and submitted to BLAST at https://www.ncbi.nlm.nih.gov/ to check their specificity. All the primers were received from (Integrated DNA Technologies, Coralville, Iowa) in lyophilized form. They were reconstituted in nuclease free water. The thermal cycling conditions used were: 10 min at 95 °C for initial activation, for PCR amplification; denaturation at 95 °C for 30 s, annealing at 60 °C for 30 s and extension at 72 °C for 30 s, for 40 cycles. The relative fold change in gene expression in treatment group, compared with untreated control was calculated as 2^−ΔΔCt^ method.

### Statistical analysis

Statistical Analysis was performed using one-way ANOVA and Bonferroni post hoc test using IBM, SPSS, Statistics software, version 21. *P-*value less than 0.05, was considered as statistically significant.

## Results

### In vitro studies

#### Effect of honey and its isolated proteins on phagocytes oxidative burst

**Fig. 2:**
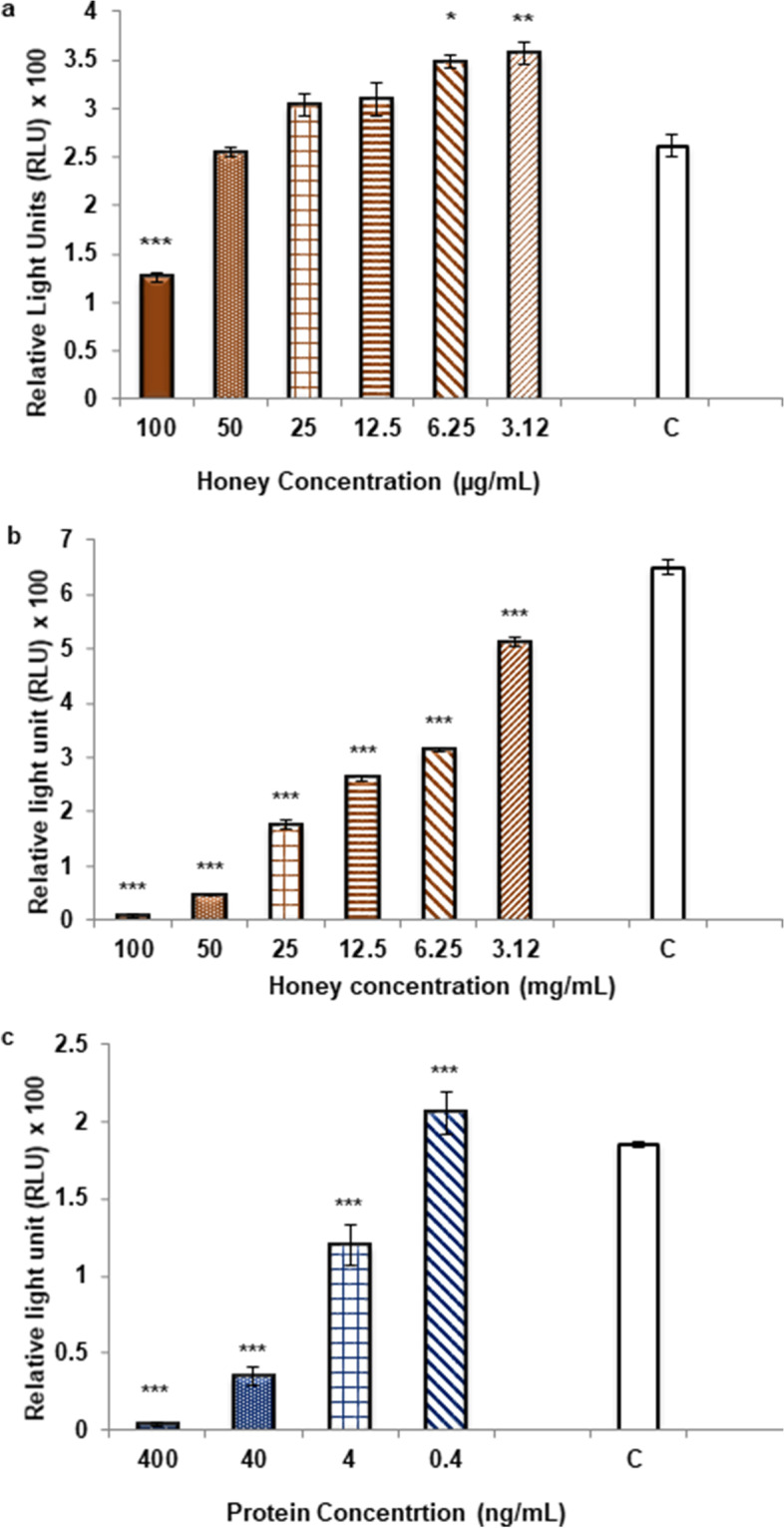
Effect of *Ziziphus* honey and its isolated protein treatment on ROS production (**a**) Treatment with low doses (3.12–100 μg/mL) of honey (**b**) Treatment with high doses (3.12–100 mg/mL) of honey (**c**) Treatment of isolated honey proteins (0.4—400 ng/mL). Results are presented in relative light units (RLU) and oxidative burst activity of whole blood using luminol as a probe. Each vertical bar represents a mean of triplicate. Error bars represent standard deviation (SD) of the means. Significance difference was compared to the control (C) where C = Cells stimulated with zymosan and *** represents the *P*-value of ≤ 0.001, ** (*P* ≤ 0.01), and * (*P* ≤ 0.05). Statistical analysis was done by using one-way ANOVA

Inhibitory effects of honey and its isolated proteins was tested against oxidative burst generated from zymosan activated human whole blood phagocytes. Honey showed dose dependent inhibitory effects on ROS. The lower doses (3.12–25 µg/mL) did not showed inhibitory effect (Fig. 2a) while the treatment with higher doses (3.12–100 mg/mL) of honey potently inhibit the ROS with an IC_50_ value of 5.98 ± 0.1 mg/mL where as 48% inhibition (*P* ≤ 0.001) was observed at 100 μg/ml concentration, while non-significant (6.3%) inhibition was shown at 50 μg/mL. (Fig. 2b). In contrast the proteins isolated from honey significantly (*P* ≤ 0.001) inhibited the ROS at much lower concentrations with an IC_50_value of 7.40 ± 1.5 ng/mL, as compared to crude honey (Fig. 2c.).

### Effect of honey and its isolated proteins on nitric oxide (NO) production

**Fig. 3:**
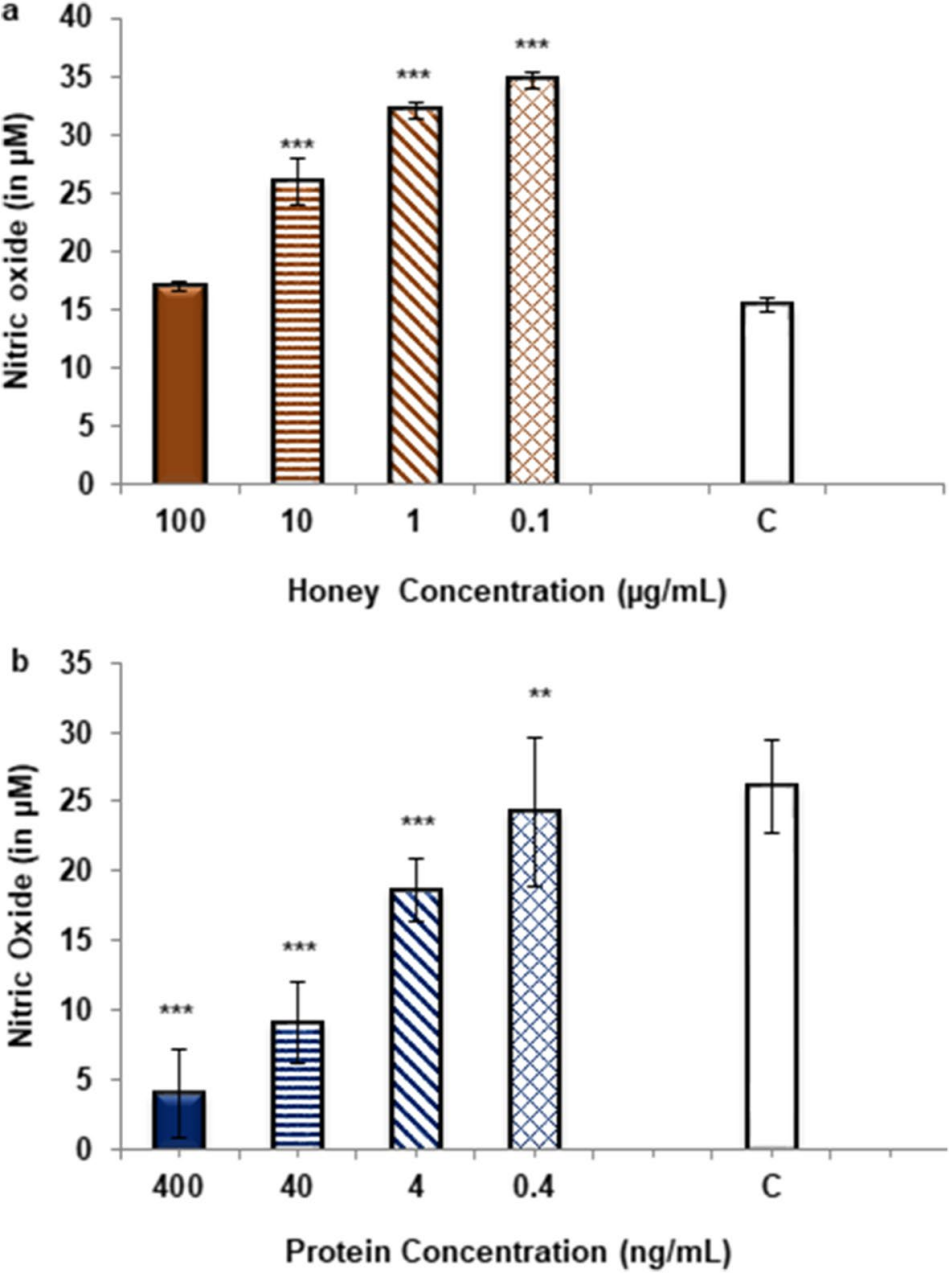
Effect on NO production (**a**) *Ziziphus* honey treatment (**b**) isolated honey proteins treatment. Data represented as mean ± S.D. of triplicates. Significance difference was compared to the control (C) where C = Cells stimulated with LPS, symbols *** and ** represents the P-values of ≤ 0.001. and ≤ 0.01 respectively. Statistical analysis was done by using One- way ANOVA

Effect of honey and its isolated proteins on NO production was determined on mouse macrophage cells J774.2 activated with bacterial lipopolysaccharide (LPS). Honey treatment significantly increased the NO production from 0.1 to 10 µg/mL concentration (P ≤ 0.001) while non-significant increase was observed at the dose of 100 µg/mL (Fig. 3a). The treatment with isolated proteins significantly inhibited the NO production at all tested concentrations (4–400 ng/mL) as compared to control with (*P* ≤ 0.001) and for 0.4 ng/ml (*P* ≤ 0.01) and with an IC_50_ value of 9.5 ± 1.3 ng/mL (Fig. 3b.).

### Cytotoxicity of isolated honey proteins

The toxicity of isolated honey proteins was evaluated on normal mouse fibroblast (NIH-3T3) (IC_50 =_ 21.7 ± 1.1 ng/mL) and normal human fibroblast BJ (IC_50 =_ 2.5 ± 0.1 µg/mL) cell lines. The toxic effect was observed at much higher concentrations compared to doses that showed anti-inflammatory effects in in vitro assays, where the IC_50_ values for inhibition of ROS and NO were found to be 7.40 and 9.5 ng/ml respectively (Table 1.).

**Table 1:** Cytotoxicity of Crude Honey Proteins: Cytotoxicity of crude honey proteins on NIH-3T3 (mouse fibroblast) and BJ (human fibroblast) cell lines by MTT assay. Results are presented as mean ± SD of triplicates

**Sample/Compound**	**MTT Cytotoxicity Assay**	
	**BJ cells** **(IC**_**50**_** ± SD µg/mL)**	**NIH-3T3 cells** **(IC**_**50**_** ± SD ng/mL)**
**Honey Crude Protein**	2.5 ± 0.1	21.7 ± 1.1

In Vivo Studies.

### Effect on blood glucose level

High blood glucose levels (≥ 200 mg/dL) were observed in Streptozotocin (STZ) induced diabetic rats. Significantly reduced (P ≤ 0.001) blood glucose levels were observed with treatment of honey as well as its isolated protein in both oral and IP routes when compared to non-treated diabetic rats group (Table. 2).

**Table 2:** Blood Glucose Level of non-diabetic and diabetic rats: Effect of Honey and its isolated protein on blood glucose level of treated and non-treated diabetic rats. where n = 6 rats/group

***Groups***	***Before Treatment*** **(mg/dL)**	***After Treatment*** **(mg/dL)**
NC	77 ± 6.4	77.4 ± 6.1
DC	318.5 ± 10.3***	559.6 ± 31.7
HO	312.1 ± 17.4***	284 ± 22.4 + + +
HI	300.5 ± 26.2***	283.6 ± 25.7 + + +
PO	315.8 ± 22.1***	290.1 ± 21.4 + + +
PI	319 ± 20.3 ***	309.1 ± 21.6 + + +
IND	314.5 ± 16.3 ***	308.1 ± 16 + + +

Where NC = Non-diabetic control, DC = Diabetic control, HO = Oral honey treatment, HI = I.P honey treatment, PO = oral proteins treatment, PI = I.P proteins treatment, I. P = Intraperitoneal route and IND = Indomethacin. ***p < 0.001compared to NC group; +  +  + *p* < 0.001 compared to DC group

### Effect on body weight of rats

**Fig. 4:**
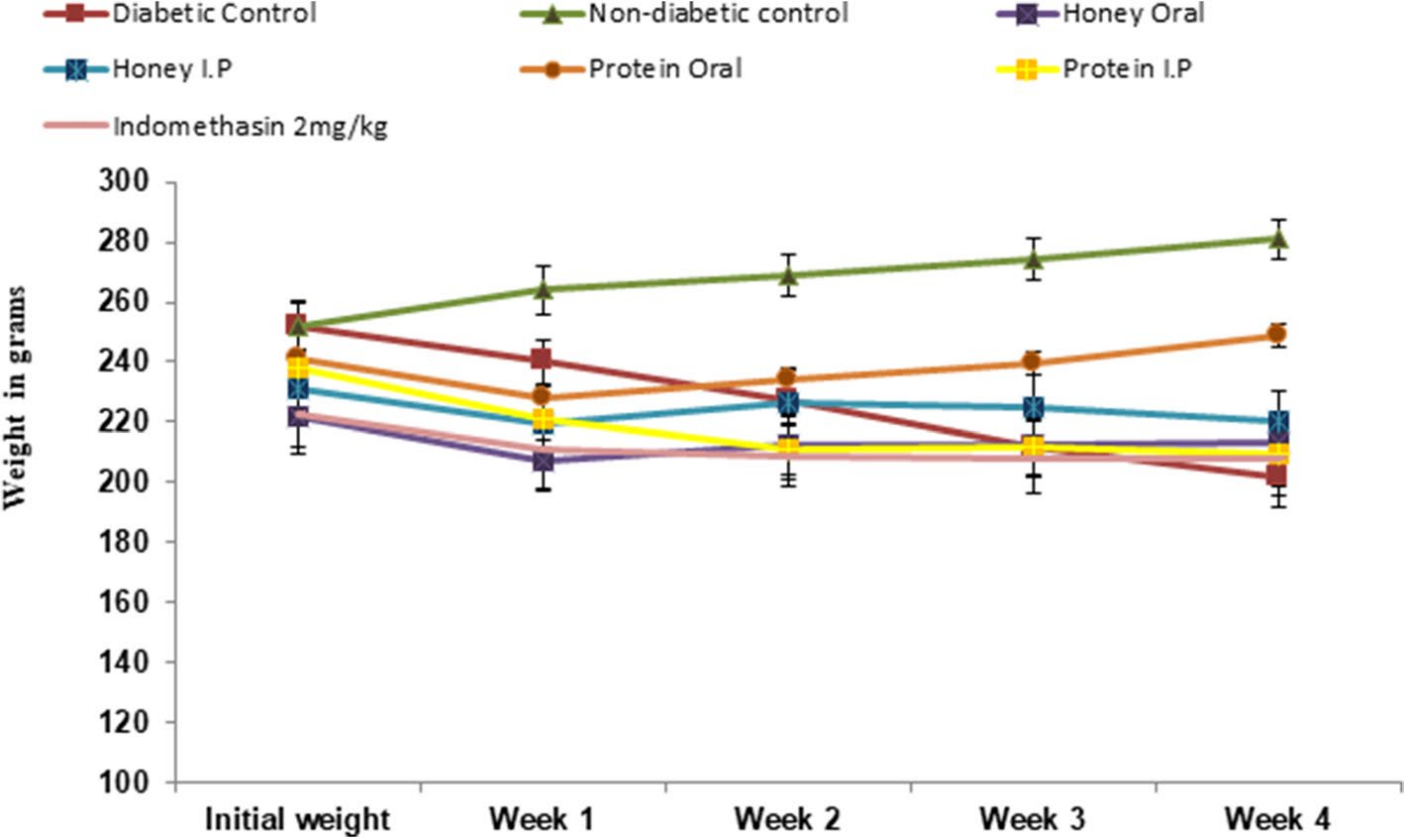
Body weight of Normal, diabetic non-treated, and diabetic treated rats on weekly basis

The body weights of normal, treated and non-treated diabetic rats were monitored weekly for one month. Reduction was observed in the body weight of diabetic control group while non-diabetic group showed an increase in body weight. Mild increase in weight was observed in both intraperitoneal and oral honey treated groups. The isolated proteins treated rats through oral route showed noticeable increase in body weights as compared to rats received treatment by IP route. Indomethacin treatment also showed little weight gain (Fig. 4). All changes in body weight throughout the experiment were found to be statistically non-significant.

### Analysis of expression of inflammatory genes on non-diabetic, treated and non-treated diabetic groups

**Fig. 5:**
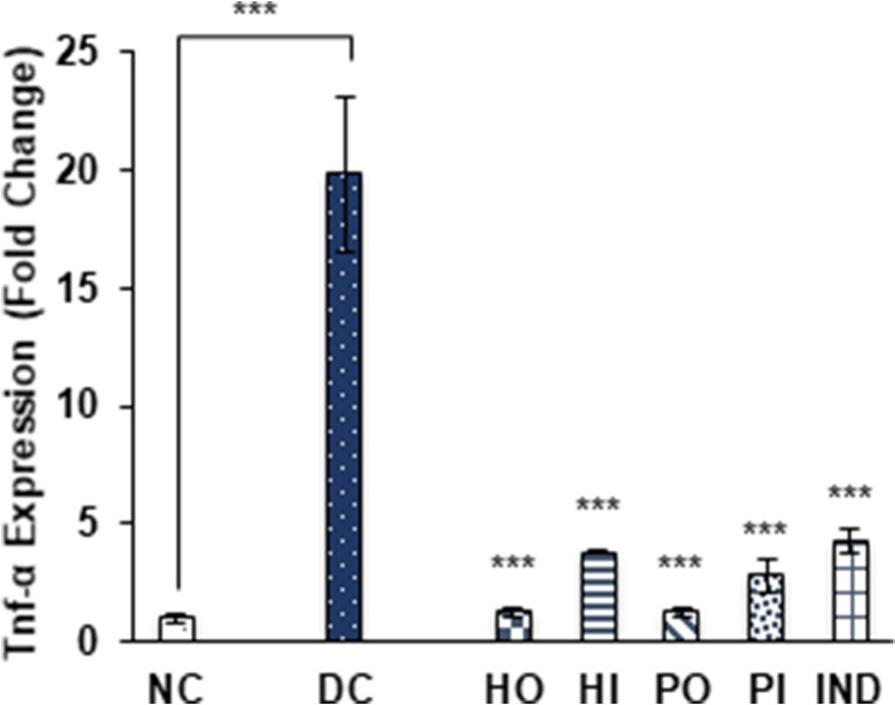
Effect of Different treatments on Tnf-α expression. Data were represented as mean ± SD, ***p < 0.001 vs Diabetic control group (DC). Statistical analysis was done by using One- way ANOVA

The mRNA expression of TNF-α was significantly (*p* ≤ 0.001) increased in STZ induced diabetic rats as compared to non-diabetic rats with 19.8 ± 3.3 fold. In comparison with non-treated diabetic group (DC) the levels of TNF-α were reduced to 1.26 ± 0.19 fold by oral honey treatment (HO) and 3.82 ± 0.06 fold by intraperitoneal (I.P) honey treatment. Both oral (PO) and I.P treated (PI) isolated protein groups lower the expression of this inflammatory marker by 1.28 ± 0.18 and 2.8 ± 0.69 fold respectively. The control drug indomethacin (IND) also reduced the TNF-α level by 4.31 ± 0.53-fold compare to non-treated diabetic rats group (Fig. 5).

**Fig. 6:**
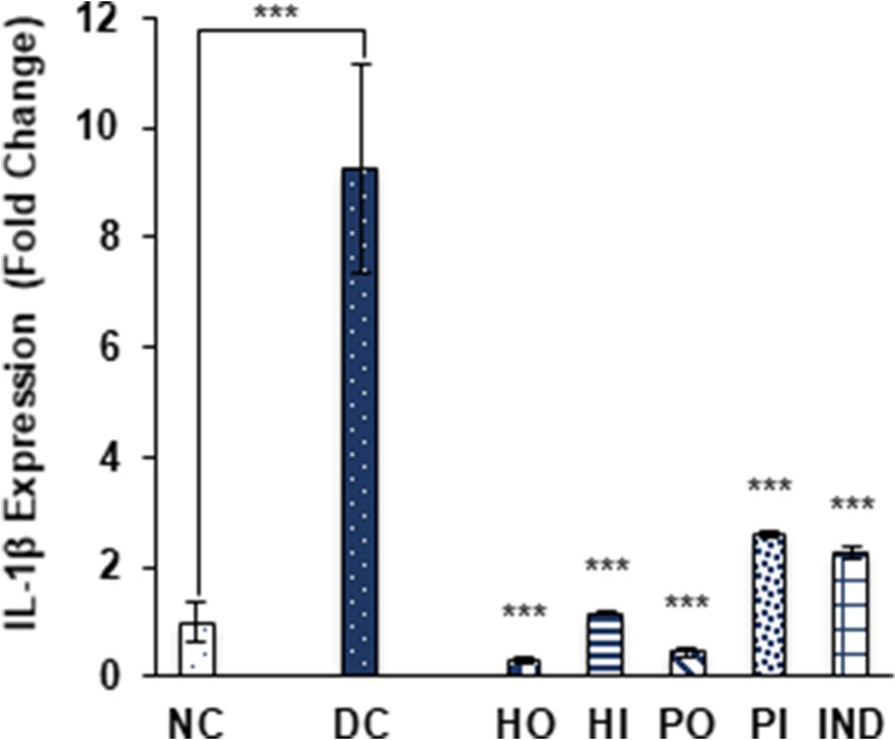
Effect of different treatments on IL-1β expression. Data were represented as mean ± SD, ***p < 0.001 vs Diabetic control group (DC). Statistical analysis was done by using One- way ANOVA

The IL-1β production was up regulated by 9.23 ± 1.89 fold in non-treated diabetic group (DC), while treatment of honey and isolated proteins by both oral (0.32 ± 0.05 and 0.46 ± 0.08 respectively) and I.P (1.17 ± 0.04 and 2.59 ± 0.07 respectively) routes down regulated the IL-1β expression. Indomethacin treatment also reduced the IL-1β expression by 2.25 ± 0.10 fold (Fig. 6).

**Fig. 7:**
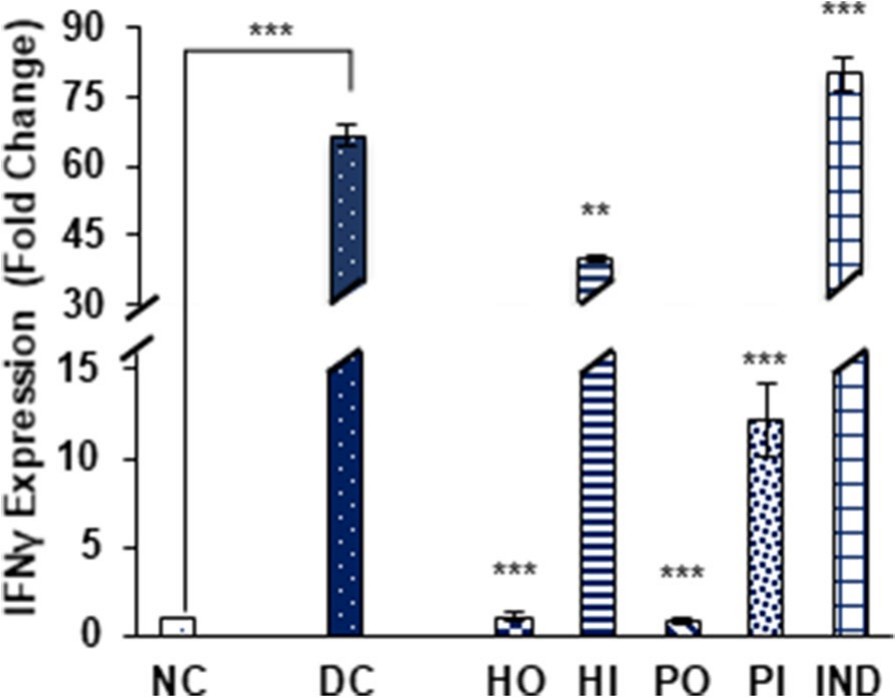
Effect of Different treatments on IFNγ expression. Data were represented as mean ± SD, **p < 0.01, ***p < 0.001 vs Diabetic control group (DC). Statistical analysis was done by using One- way ANOVA

The IFN-γ expression was augmented by 66.58 ± 2.4 fold in diabetic rats as compared to non-diabetic rats. The treatment of indomethacin boosted the expression of IFN-γ significantly by 79.89 ± 3.8 fold. The significant reduction in the expression of this cytokine was observed in oral honey and proteins (1.02 ± 0.2 and 0.88 ± 0.1 fold respectively) treated groups and in I.P proteins (12.16 ± 2.02 fold) and honey I.P (39.9 ± 0.9 fold) treated groups (Fig. 7).

**Fig. 8:**
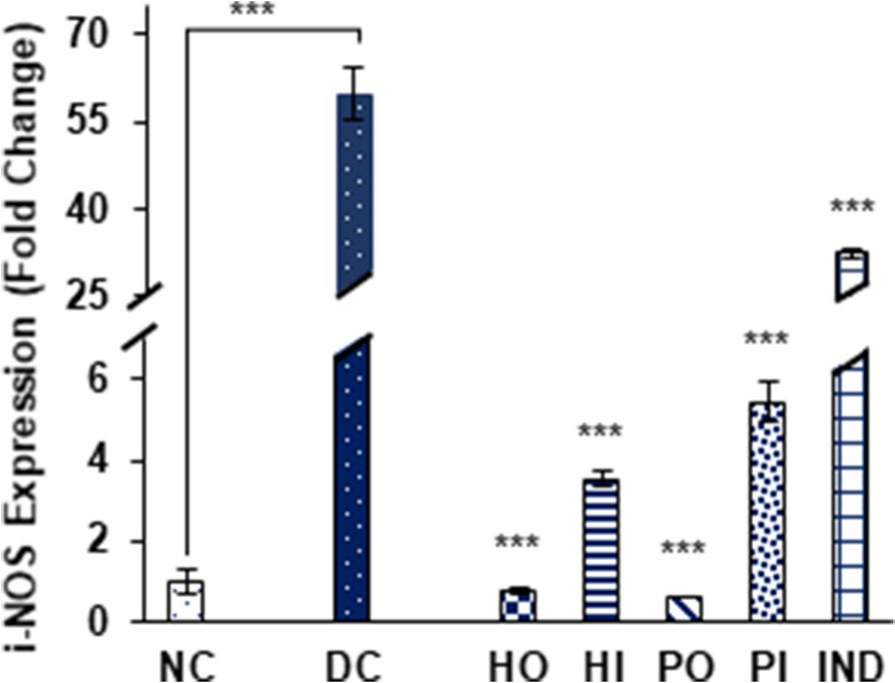
Effect of Different treatments on i-NOS expression. Data were represented as mean ± SD, ***p < 0.001 vs Diabetic control group (DC). Statistical analysis was done by using One- way ANOVA

Expression of inducible nitric oxide synthase (iNOS) was efficiently up regulated in non-treated diabetic group by 59.83 ± 4.6 fold, while significant reduction in expression was observed in all treated groups including honey oral and I.P (0.78 ± 0.06 and 3.54 ± 0.21 respectively), Protein oral and I.P (0.62 ± 0.02 and 5.45 ± 0.5 respectively) and indomethacin treated group by 32.32 ± 0.7 fold (Fig. 8).

**Fig. 9:**
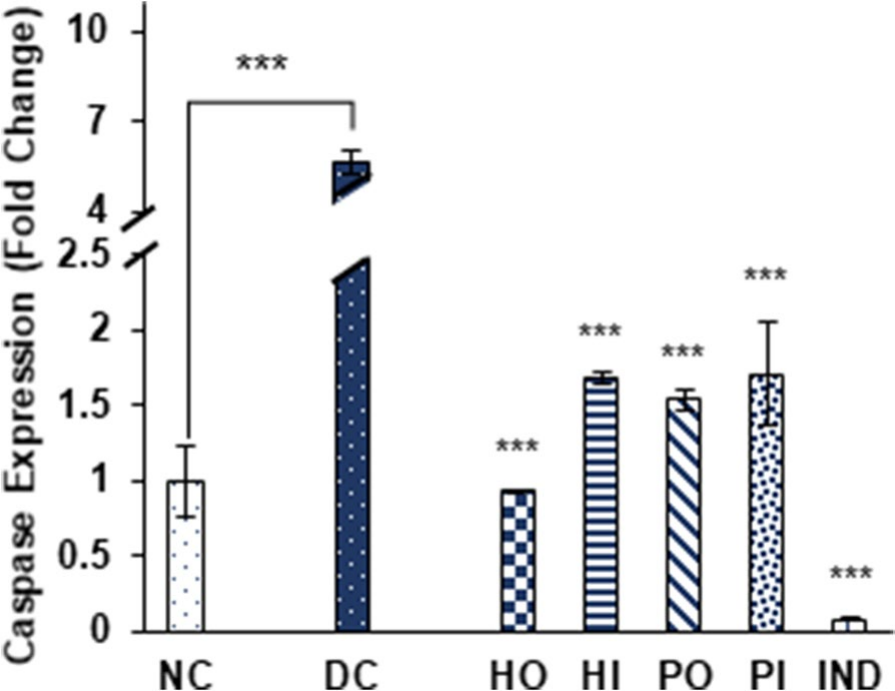
Effect of Different treatments on Caspase expression. Data were represented as mean ± SD, ***p < 0.001 vs Diabetic control group (DC). Statistical analysis was done by using One- way ANOVA

Honey and its isolated protein suppresses the expression of caspase-1 by 0.93 ± 0.01 and 1.54 ± 0.06 fold through oral and 1.68 ± 0.04 and 1.71 ± 0.34 fold by I.P route respectively. Indomethacin decreases the expression of caspase-1 by 0.08 ± 0.01 fold (Fig. 9).

**Fig. 10:**
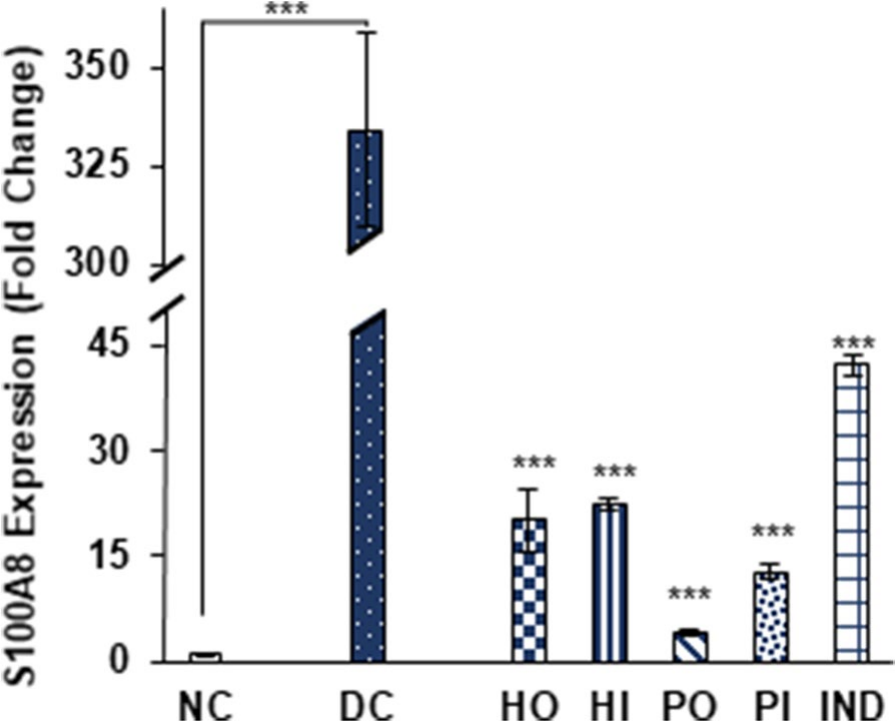
Effect of Different treatments on S100A8 expression. Data were represented as mean ± SD, ***p < 0.001 vs Diabetic control group (DC). Statistical analysis was done by using One- way ANOVA

**Fig. 11:**
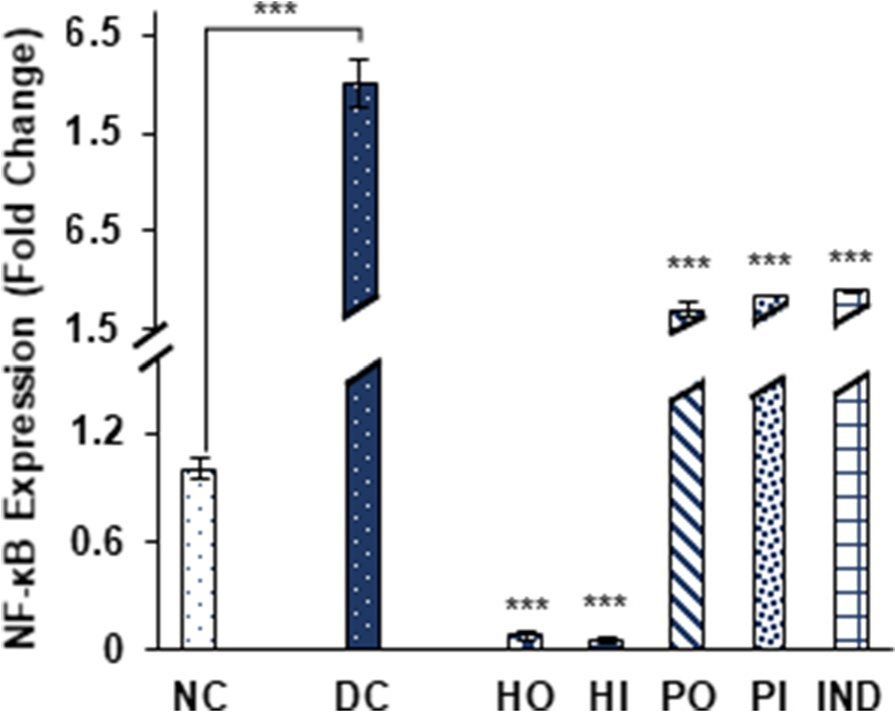
Effect of Different treatments on NF-κB expression. Data were represented as mean ± SD, ***p < 0.001 vs Diabetic control group (DC). Statistical analysis was done by using One- way ANOVA

S100A8 and NF-κB expression were also suppressed significantly in response to honey, isolated protein and indomethacin. Honey oral and I.P treatment reduced S100A8 by 20.1 ± 4.6, 22.53 ± 0.92 respectively and honey protein oral and I.P treatments by 4.21 ± 0.4 and 12.8 ± 1.04 fold respectively while 42.19 ± 1.3-fold reduction in expression was observed by indomethacin treated group (Fig. 10). In case of NF-κB significant suppression was observed in honey oral; 0.08 ± 0.01, honey I.P; 0.06 ± 0.01, protein oral; 4.47 ± 1.2, protein I.P; 6.6 ± 0.08 and indomethacin treated group 7.24 ± 0.3. (Fig. 11). The over expression of caspase 1, S100A8 as well as NF-κB was observed by 5.63 ± 0.37, 334 ± 24.6 and 39.07 ± 3.4 fold respectively in non-treated diabetic group as compared to non-diabetic group (Fig. 9,10,11). Results represented in 2-ΔΔct as mean ± S.D. fold change of triplicate after HPRT (Hypoxanthine–guanine phosphoribosyl transferase) normalization.

## Discussion

New therapeutic approaches targeting inflammation have been developed by using naturally obtained bioactive compounds from natural products to cure various diseases [34]. Bioactive components of honey including proteins, amino acids, ascorbic acid, organic acids, vitamins and trace elements [35] tends to have immunomodulatory activity [18, 19]. We are extensively involved in exploring the immunomodulatory and therapeutic potential of honey proteins. Previously the characterization and anti-inflammatory effect of *Zizphus* honey and its isolated proteins were reported by our colleagues, where separated protein peaks also showed anti-inflammatory effects [21]. The similar observation here with fresh *Ziziphus* honey and isolated protein samples further validate it’s in vitro anti-inflammatory effects. Here in we chose the isolated crude honey protein to ensure the availability of good amount to conduct in vivo studies and to compare the therapeutic effect of total honey protein with raw honey in STZ induced diabetic rats by targeting inflammatory markers involved in diabetic pathogenicity.

The damage caused due to unbalance levels of oxidants and antioxidants or by gross production of reactive oxygen species initiate stress condition in the body, which can be healed by honey being having anti-inflammatory effect [36, 37]. In present study proteins isolated from *Ziziphus* honey showed potent inhibitory effect on reactive oxygen species (ROS) generated from whole blood phagocytes at very low concentrations (ng/ml) (Fig. 2c) as compared to crude honey, which showed dose dependent immunomodulation. Inhibition of phagocyte oxidative burst was shown by *Ziziphus* honey at higher concentrations (mg/ml) (Fig. 2b) whereas at lower doses (μg/ml) stimulatory effect was observed (Fig. 2a). Previously the proteins were isolated and characterized from seven different types of honey including acacia, thyme, pine, china berry, gum tree, citrus and honey from local areas of Sindh, Pakistan and were evaluated for their anti-inflammatory potential where they found to suppress oxidative burst at very low concentrations [38]. In present study the proteins from *Ziziphus* honey also showed similar inhibitory potential on production of ROS from phagocytes at very low doses, which indicates that the anti-oxidant effect of honey proteins is independent of the source and type of honey. Nitric oxide (NO), is a key regulator of immune system and its high levels are involved in various inflammatory and autoimmune disorders. Isolated honey proteins significantly inhibited the NO production at very low concentrations (IC_50_ 9.5 ± 1.3 ng/ml) (Fig. 3b) on the other hand crude honey at lower doses (up to 100 µg/mL) showed no inhibitory effect on NO (Fig. 3a). Lipopolysaccharide (LPS) induces an immune response in macrophages. It is an outer bacterial membrane component which by binding to CD14 and Toll-like receptors, activates the transcription factor NF-κB that regulates various other genes involved in apoptosis, cell proliferation, cell survival and inflammation [39, 40]. Down regulation of NF-κB gene or interruption of downstream signaling pathways might be responsible for inhibition of NO by honey proteins [41, 42]. The toxic effect of honey protein was determined on two different cell lines of normal origin including human (BJ) and mouse (NIH-3T3) where the observed effects were far beyond the concentrations that showed anti-inflammatory effects (Table 1).

Destruction in pancreatic β-cells caused by streptozotocin creates insulin deficiency or hypoinsulinemia [43] resulted in hyperglycemic condition. In present study *Ziziphus* honey and its isolated protein by both oral and I.P routes significantly reduced fasting blood glucose level compared to non-treated diabetic rats (Table 2). Previously the effect of honey on glycemic control was reported in alloxan and fructose induced diabetic rats [27]. The anti-diabetic effect of honey in noise induced hyperglycemic rats and isolated proteins in streptozotocin inducedwas also reported [25, 45]. Fructose, the main sugar in honey being an activator of glucokinase was shown to be responsible for the hypoglycemic effect of honey [44]. However, this hypoglycemic effect of honey was not observed in non-diabetic individuals [45] suggesting that insulin doesn’t respond to fructose in lower glucose concentrations [46]. Present study suggests that not only fructose but proteins in honey are also responsible for lowering blood glucose levels with unknown mechanism. The observed decrease in blood glucose level by anti-inflammatory drug indomethacin in diabetic group rats suggest that the hypoglycemic effect shown by honey and its isolated protein may also be due to their anti-inflammatory effect [47], as inflammatory mediators also induces hyperglycemia [48]. Improvement in body weight was observed in diabetic rats receiving oral honey and isolated proteins as compared to non-treated diabetic rats (Fig. 4), where continuous reduction in body weight was observed. No effect on body weight was shown by diabetic rats receiving I.P treatment of honey and its isolated protein, validating the hormone regulation and appetite modulatory effects of honey as well as its protein via oral route [49].

Various inflammatory processes are involved in the complications related to diabetes [6]. NF-κB is an important transcriptional factor belongs to Rel family which is responsible for the expression of iNOS and various cytokines including TNF-α, and IL-1β [50]. In diabetes the cytokines including interferon-γ (IFN-γ) and interleukin 1 (IL-1β) are responsible to mediate an autoimmune response by activating NF-κB which causes destruction in pancreatic β-cells. Cellular dysfunction and apoptosis is induced by inflammatory mediators which modulate the expression of several related genes. Modifications in the expression of more than sixty-six genes by these cytokines were reported. Which were mostly initiated by NF-κB [51]. Cytokine induced apoptosis can be prevented in pancreatic β-cells by inhibiting NF-κB pathway, posing a potent therapeutic strategy for pancreatic β-cells protection [52]. The association of hyperglycemia and IFN-γ was also reported [53].

The inflammatory mediators TNF-α, IL-1β, IFN-γ, iNOS and NF-κB have close association with progression of diabetes. Down regulation of NF-κB, as well as other selected inflammatory markers genes, suggest that honey and its protein has strong tendency to inhibit inflammation associated with diabetes (Fig. 5, 6, 7, 8 and 11). Previously, the suppression of inflammatory markers including iNOS, TNF-α, IL-1β and NF-κB were reported in honey treated RAW264.7 macrophages [54]. In various inflammation associated diseases, including diabetes mellitus, the levels of S100A8 (Calgranulin A), a small calcium binding protein also known as MRP8, used as a biomarker for diagnostic purpose owing to its secretion in a disease-specific manner [55]. Elevated levels of S100A8 expression in diabetic wounds were reported [56]. Also the insulin secretion in MIN6 K8 β-cells was inhibited in the presence of S100A8 [57]. Caspase-1 is known to be a key player in innate immunity belongs to the sub family of inflammatory caspases. It activates the inactive forms of pro-inflammatory cytokines IL-1β and IL-18 [58, 59], the over expression of these cytokines promotes several diseases including diabetes mellitus. The significant down regulation of both S100A8 and caspase1 was observed in all groups treated with *Ziziphus* honey and its isolated proteins (Fig. 9 and 10). The isolated honey proteins at very low concentration showed therapeutic potential in STZ induced diabetic rats superior to the crude honey.

## Conclusion

The present work revealed the dose dependent immunomodulatory potential of both honey and its isolated proteins by suppressing phagocyte oxidative burst with an IC_50_ values of 5.98 mg/mL and 7.4 ng/mL respectively, inflammatory markers and inhibition of nitric oxide by isolated proteins with an IC_50_ value of 9.5 ng/mL. The higher doses of honey are required to achieve the similar therapeutic effects that were obtained in small doses of isolated proteins. Both honey and protein showed hypoglycemic effect by lowering blood glucose levels when given orally as well as through intraperitoneal route. The potent suppression of inflammatory markers TNF-α, IL-1β, IFN-γ, iNOS, caspase 1, Calgranulin A (S100A8) and NF-κB by honey and its isolated proteins in diabetic rats revealed ceasing of generalized inflammation. Due to its high effectiveness at lower doses, protein treatment is anticipated to be more convenient. Our results are in the primary stages and further clinical studies on honey proteins are required to evaluate the therapeutic effects of honey proteins in various metabolic disorders related to inflammation including diabetes mellitus.

## Data Availability

The datasets generated during the current study are available from the corresponding author on reasonable request.

## Abbreviations

DM: Diabetes Mellitus
ROS: Reactive oxygen species
NO: Nitric oxide
MTT: (3-[4,5-Dimethylthiazol-2-yl]-2,5-diphenyl tetrazolium bromide) assay
STZ: Streptozotocin
IP: Intraperitoneal
TNF-α: Tumour necrosis factor α
IL-1β: Interleukin-1 beta
IFN-γ: Interferon gamma
iNOS: Inducible nitric oxide synthase
NF-κB: Nuclear factor-κB
HPRT: Hypoxanthine phosphoribosyl transferase 1
IDF: International Diabetes Federation
MRJP1: Major royal jelly protein 1
RLU: Relative light unit
ng: Nanogram
mg: Milligram
mL: Milliliter
mg: Microgram
LPS: Lipopolysaccharides
CO_2_: Carbondioxide
DMSO: Dimethyl sulfoxide
DC: Diabetic Control
HO: Honey Oral
HI: Honey Intraperitoneal
PO: Protein oral
PI: Protein Intraperitoneal
IND: Indomethacin
RNA: Ribonucleic acid
cDNA: Complementary DNA
PCR: Polymerase chain reaction
qRT PCR: Real-Time Quantitative Reverse Transcription PCR
BLAST: Blast Basic Local Alignment Search Tool
ANOVA: Analysis of variance
SPSS: Statistical Package for the Social Sciences
